# Tenogenic induction of human adipose-derived stem cells by soluble tendon extracellular matrix: composition and transcriptomic analyses

**DOI:** 10.1186/s13287-022-03038-0

**Published:** 2022-07-29

**Authors:** Ying Rao, Chenxian Zhu, Hoi Ching Suen, Shuting Huang, Jinyue Liao, Dai Fei Elmer Ker, Rocky S. Tuan, Dan Wang

**Affiliations:** 1 Institute for Tissue Engineering and Regenerative Medicine, The Chinese University of Hong Kong, Sha Tin, Hong Kong, SAR China; 2 School of Biomedical Sciences, Faculty of Medicine, The Chinese University of Hong Kong, Sha Tin, Hong Kong, SAR China; 3 Department of Orthopaedics and Traumatology, The Chinese University of Hong Kong, Sha Tin, Hong Kong, SAR China; 4 Ministry of Education Key Laboratory for Regenerative Medicine, The Chinese University of Hong Kong, Sha Tin, Hong Kong, SAR China; 5 Department of Chemical Pathology, Faculty of Medicine, The Chinese University of Hong Kong, Sha Tin, Hong Kong, SAR China; 6 Center for Neuromusculoskeletal Restorative Medicine, Hong Kong Science Park, Sha Tin, Hong Kong, SAR China

**Keywords:** Tendon, Extracellular matrix, Adipose-derived stem cells, Mass spectrometry, RNA sequencing, Bioinformatics

## Abstract

**Background:**

Tendon healing is clinically challenging largely due to its inferior regenerative capacity. We have previously prepared a soluble, DNA-free, urea-extracted bovine tendon-derived extracellular matrix (tECM) that exhibits strong pro-tenogenic bioactivity on human adipose-derived stem cells (hASCs). In this study, we aimed to elucidate the mechanism of tECM bioactivity via characterization of tECM protein composition and comparison of transcriptomic profiles of hASC cultures treated with tECM versus collagen type I (Col1) as a control ECM component.

**Methods:**

The protein composition of tECM was characterized by SDS-PAGE, hydroxyproline assay, and proteomics analysis. To investigate tECM pro-tenogenic bioactivity and mechanism of action, differentiation of tECM-treated hASC cultures was compared to serum control medium or Col1-treated groups, as assessed via immunofluorescence for tenogenic markers and RNA Sequencing (RNA-Seq).

**Results:**

Urea-extracted tECM yielded consistent protein composition, including collagens (20% w/w) and at least 17 non-collagenous proteins (< 100 kDa) based on MS analysis. Compared to current literature, tECM included key tendon ECM components that are functionally involved in tendon regeneration, as well as those that are involved in similar principal Gene Ontology (GO) functions (ECM-receptor interaction and collagen formation) and signaling pathways (ECM-receptor interaction and focal adhesion). When used as a cell culture supplement, tECM enhanced hASC proliferation and tenogenic differentiation compared to the Col1 and FBS treatment groups based on immunostaining of tenogenesis-associated markers. Furthermore, RNA-Seq analysis revealed a total of 584 genes differentially expressed among the three culture groups. Specifically, Col1-treated hASCs predominantly exhibited expression of genes and pathways related to ECM-associated processes, while tECM-treated hASCs expressed a mixture of ECM- and cell activity-associated processes, which may explain in part the enhanced proliferation and tenogenic differentiation of tECM-treated hASCs.

**Conclusions:**

Our findings showed that urea-extracted tECM contained 20% w/w collagens and is significantly enriched with other non-collagenous tendon ECM components. Compared to Col1 treatment, tECM supplementation enhanced hASC proliferation and tenogenic differentiation as well as induced distinct gene expression profiles. These findings provide insights into the potential mechanism of the pro-tenogenic bioactivity of tECM and support the development of future tECM-based approaches for tendon repair.

**Supplementary Information:**

The online version contains supplementary material available at 10.1186/s13287-022-03038-0.

## Introduction

Tendon is a fibrous band of collagenous tissue that connects muscle to bone and functions in force transmission during musculoskeletal movement [1]. Tendon injuries and diseases carry significant morbidity and are estimated to account for 45% of the more than 32 million musculoskeletal injuries in the USA each year [2]. These injuries have a prolonged recuperation period because of the intrinsically poor natural healing response of tendon tissues due to low cellularity and low vascularity [3]. Current clinical treatments for small tendon injuries can usually restore tissue integrity, but full functionality is rarely attained [4]. On the other hand, large-to-massive tendon injuries entail high re-rupture rates and various complications can persist several years postinjury [5]. Within this context, numerous growth factors have been utilized for tendon repair, including fibroblast growth factors (FGF), platelet-derived growth factors (PDGF), insulin-like growth factors (IGF), transforming growth factor-beta (TGF-*β*), bone morphogenetic protein 2 (BMP2) and connective tissue growth factors (CTGF) [6, 7]. In addition to these growth factors, extracellular matrix (ECM)-based approaches have also been widely studied and utilized in clinical practice and biomedical research for tendon repair [8]. The ECM is a complex three-dimensional network of interacting macromolecules that occupies the space between cells and is principally responsible for both force transmission and tissue structure maintenance [9]. The ECM is also unique in its tissue-specific bioactivity because each tissue or organ contains a unique ECM composition that contributes to tissue-specific structure and function [9, 10]. Indeed, a number of ECM-based, Food and Drug Administration (FDA)-approved biomaterials, such as GraftJacket^™^ (Wright Medical Group), Zimmer^®^ Collagen Repair Patch (Zimmer, Inc.) and TissueMend^®^ (Stryker), have shown promising tendon healing potential.

When developing ECM-based biomaterials for therapeutic use, a major issue is that their bioactive properties can differ due to batch-to-batch preparations. Such variability can be caused by a variety of reasons, including donor age and health, tissue source, storage conditions and decellularization methods [11]. There are many methods to extract tissue-derived ECM for clinical application, with acid-pepsin digestion being one of the classic techniques [10]. During the acid solubilization and/or pepsin digestion process, slight changes such as differences in pH or ionic concentration can lead to inconsistencies in ECM quality [12]. Additionally, tissue ECM produced by acid-pepsin solubilization, such as some of the commercial ECM scaffolds mentioned above, is composed primarily of collagens. For example, GraftJacket^™^ is an acellular human dermal collagen matrix, which has been shown to provide functional support and reinforcement of tendon/ligament tissue. TissueMend^®^, which is designed to reinforce the tendon during repair and facilitate tissue remodeling, is also a collagen matrix-based material derived from fetal bovine skin. Although tendon ECM is predominantly composed of collagens, which account for around 60–85% of its dry weight [13], its non-collagenous ECM components, such as proteoglycans, glycoproteins and glycoconjugates, also play important biological roles, including collagen fibril formation and tenocyte homeostasis that further contribute to overall tendon function and repair [13, 14]. However, details of the organization and hierarchical locations of these non-collagenous ECM components are generally less well understood. In addition, the activities of tendon ECM-sequestered biofactors must also be taken into consideration [13]. Therefore, further work on deciphering the composition and the mechanism of action of tendon ECM will be needed to elucidate the issues of donor-to-donor variability and subsequently engineer well-defined, bioactive ECM-based products.

To improve the yield of non-collagenous components during tendon ECM extraction, our laboratory has previously developed a urea-based method to prepare a soluble, DNA-free, ECM fraction from bovine tendons (tECM), which exhibited strong pro-tenogenesis effects on human adipose-derived stem cells (hASCs) [15]. In this study, to further investigate the mechanism of action of tECM bioactivity, we characterized the protein compositions of tECM by sodium dodecyl sulfate-polyacrylamide gel electrophoresis (SDS-PAGE) analysis, hydroxyproline assay and mass spectrometry (MS)-based proteomics analysis. Subsequently, we compared the tenogenic activity of hASCs upon exposure to tECM with that in the presence of collagen type I (Col1), a major tendon ECM component, on the basis of established tenogenesis-associated markers detected by immunofluorescence staining. In addition, RNA sequencing (RNA-Seq) and bioinformatics analysis were applied to characterize the transcriptomic effects of tECM on hASCs.

## Materials and methods

### tECM extraction

tECM was extracted as described previously [15]. Briefly, bovine Achilles tendons (2- to 3-month-old calves from Research 87, USA) were harvested, cryosectioned at 10 µm thickness (Cryotome, Thermo Fisher Scientific, USA) and decellularized using 1% Triton X-100 (Sigma-Aldrich, USA) and treatment with DNase (200 U/mL, Worthington, USA), and RNase (50 U/mL, Worthington). The decellularized tissue was extracted using 3 M urea (Sigma-Aldrich) at 4 °C for 3 days and then dialyzed (2 K molecular weight cutoff, Thermo Fisher Scientific) against phosphate-buffered saline (PBS, Santa Cruz Biotechnology, USA) at 4 °C for 2 days. The dialysate was then spin-concentrated using protein centrifugation tubes (3 K molecular weight cutoff, Thermo Fisher Scientific) and stored at − 80 °C for subsequent experimental use. Prior to supplementation in culture medium, the tECM solution was filter-sterilized using a 0.22-μm polyvinylidene fluoride (PVDF) syringe filter unit (Merck Millipore, USA).

### Hydroxyproline assay

Collagen concentration in tECM was estimated using a chloramine-T hydroxyproline assay [10] and standardized with commercial bovine collagen solution (Advanced BioMatrix, Inc., USA).

### SDS-PAGE and in-gel trypsin digestion

tECM preparations were subjected to SDS-PAGE in 8% gel as previously described [16]. Based on calibration using molecular weight markers, Coomassie Blue-stained gel bands below 100 kDa were excised and processed using the In-Gel Tryptic Digestion Kit (Thermo Fisher Scientific) for peptide extraction according to the manufacturer’s protocol. Briefly, after destaining with 2 mg/mL ammonium bicarbonate in 50% (v/v) acetonitrile (ACN) at 37 °C, gel pieces were processed for alkylation reduction using 50 mM Tris [2-carboxyethyl] phosphine (TCEP) and 500 mM iodoacetamide (IAA). The gel pieces were then ACN-dehydrated and rehydrated with 50 μL trypsin (10 ng/μL). After digestion at 30 °C overnight, the tryptic peptide solution was vacuum-dried.

### MS analysis and protein identification

Nanoflow liquid chromatography (NanoLC)-matrix-assisted laser desorption/ionization (MALDI)-time of flight (TOF)/TOF mass spectrometry was performed on an Ultimate^™^ 3000 RSLCnano System (Thermo Fisher Scientific) connected to a MALDI TOF/TOF mass spectrometer (UltrafleXtreme, Bruker Daltonics, USA). The peptide mixtures from the in-gel digestion procedure were analyzed and the whole MALDI-TOF/TOF system was controlled using the HyStar 3.2 software (Bruker Daltonics). Protein categorization was performed using annotations from The Matrisome Project (In silico Matrisome, http://matrisomeproject.mit.edu) [17] and the Protein ANalysis THrough Evolutionary Relationships (PANTHER) classification system (v. 16.0, http://pantherdb.org) [18]. Gene ontology (GO), signaling pathway and protein network analyses of identified proteins were performed using the Search Tool for Retrieval of Interacting Genes/Proteins database (STRING, v. 11.5, https://string-db.org/) [19] (see Additional file 1 for experimental details).

### hASC isolation, characterization and differentiation

hASCs were isolated from the infrapatellar fat pad surgical tissue waste of patients undergoing total knee replacement surgery in accordance with The Chinese University of Hong Kong Institutional Review Board approval and guidelines as described previously [16]. Briefly, isolated cells were further sorted by BD FACSAria^™^ Fusion Flow Cytometers (BD Biosciences, USA) using the BD Biosciences human mesenchymal stem cells (MSC) analysis kit and characterized by colony-forming unit-fibroblast (CFU-F) as well as tri-lineage differentiation assays (osteogenesis, adipogenesis and chondrogenesis) as previously described [16]. hASCs at passages 4–7 were used for all experiments. To test the effect of tECM, hASCs (1 × 10^4^ cells/cm^2^) were first serum-starved in Dulbecco's modified Eagle's medium (DMEM, Gibco, USA) overnight and then cultured in basal medium, consisting of DMEM, containing 2% (v/v) fetal bovine serum (FBS, Gibco), 1% (v/v) penicillin/streptomycin (P/S, Gibco), 1% (v/v) insulin-transferrin-selenium ethanolamine (ITS-X, Gibco) and 1% (v/v) 5 mg/mL ascorbic acid (Sigma-Aldrich), supplemented with either Col1 (2% and 10% v/v of 1 mg/mL Col1 solution) or tECM (10% v/v of 1 mg/mL tECM solution) for the indicated time periods.

### Immunofluorescence and F-actin staining

Immunofluorescence and F-actin staining was performed as described previously with minor modifications [16]. At designated time points, hASCs were fixed in 4% (w/v) paraformaldehyde (Sigma*-*Aldrich) at room temperature for 15 min or 100% ice-cold methanol (Duksan Chemical Co. Ltd., Korea) at 4 °C for 10 min and then permeabilized with 0.1% (v/v) Triton-X100 or 0.5% (w/v) Saponin (Sigma-Aldrich). Blocking was done with 10% (v/v) donkey serum (Merck Millipore) in PBS. Primary antibodies used included rabbit anti-human scleraxis (SCX, 5 μg/mL, Abcam, USA), mouse anti-human tenascin C (TNC, 5 μg/mL, Abcam), rabbit anti-human collagen type I (COL1, 5 μg/mL, Abcam) and Phalloidin-iFluor 555 (1 μg/mL, Abcam), diluted in 1% (w/v) bovine serum albumin (BSA, Sigma-Aldrich) according to manufacturer’s instruction, and incubation was done at 4 °C overnight. For secondary antibodies, Alexa Fluor 488-conjugated donkey anti-rabbit IgG or Alexa Fluor 647 donkey anti-mouse IgG (Invitrogen, USA) was used after dilution to 10 μg/mL in 1% BSA, and incubation was done at room temperature for 1 h. Cells were stained for F-actin using Alexa Fluor 555-conjugated phalloidin (Thermo Fisher Scientific; 1:1000), with nuclear counter staining done using DAPI (4′,6-diamidino-2-phenylindole; 1 μg/mL, Life Technologies, USA).

Images were digitally captured using the Olympus IX83 microscope (Olympus, Japan). For each group, 3–4 samples were randomly chosen and thereafter, 2–3 images for each sample were randomly selected to quantify fluorescence intensity and coverage via NIH ImageJ as described previously [16]. Briefly, cell counts were computed by measuring the number of DAPI-positive cell nuclei. Nuclear fluorescence intensity was calculated as the mean fluorescence intensity within the cell nuclear area per field (30–40 nuclei were randomly selected per image). Fluorescence coverage was determined by measuring the percentage of fluorescently labeled area per image. Cell counts, nuclear fluorescence intensity and fluorescence coverage were statistically compared among groups using one-way ANOVA with Tukey post hoc tests.

### RNA-Seq analysis

RNA-Seq was performed using Illumina’s next-generation sequencing workflow [20]. Briefly, complementary DNA (cDNA) libraries from cell differentiation were obtained from hASCs cultured for 6 days, including the following groups: (1) control FBS group, basal medium; (2) Col1 group, basal medium with 10% (v/v) 1 mg/mL Col1 solution; and (3) tECM group, basal medium with 10% (v/v) 1 mg/mL tECM solution. RNA was isolated and library construction was performed as described previously [20]. Briefly, cellular RNA was isolated, reverse-transcribed into cDNA, purified using AMPure XP beads (Beckman Coulter, USA) and PCR-amplified for RNA-Seq library construction using TruePrep DNA Library Prep Kit V2 for Illumina (Vazyme, China) in accordance with the manufacturer’s protocols. The average full length of the library was around 450 bp, and the purified library was stored at − 20 °C until further analysis. Before RNA sequencing analysis, cDNA library quality was assessed as previously described [21].

Samples were sequenced by the Novaseq 6000 system (Illumina, USA) using approximately 150 base-pair paired-end RNA-Seq technology with 60–90 million reads per sample. Genes with > twofold change and false discovery rate (FDR) < 0.05 were considered as differentially expressed genes (DEG). GO analysis was performed using the PANTHER classification system [22] (http://geneontology.org). Pathway analysis was performed by using the Kyoto Encyclopedia of Genes and Genomes (KEGG) in the Database for Annotation, Visualization and Integrated Discovery [23] (DAVID, Resources 6.8, https://david.ncifcrf.gov). Gene set enrichment analysis (GSEA; v 4.1.0, https://www.gsea-msigdb.org/gsea/index.jsp) was performed to examine the significantly enriched KEGG pathways [24] (see Additional file 1 for experimental details).

### Quantitative PCR (qPCR) assay

To validate transcriptome profiles, qPCR was performed as described previously with minor modifications [16]. At the indicated time points, cellular RNA was isolated using a Monarch^®^ Total RNA Miniprep Kit (New England Biolabs, USA) and reverse transcribed into cDNA using LunaScript^®^ RT SuperMix Kit (New England Biolabs) with 100 ng RNA. qPCR was performed using Luna^®^ Universal qPCR Master Mix (New England Biolabs) on a QuantStudio 7 Flex Real-Time PCR system (Applied Biosystems, USA) according to the manufacturers’ instructions. Relative expression of each target gene was calculated using the ΔΔCT method and normalized to glyceraldehyde 3-phosphate dehydrogenase (GAPDH) mRNA expression. All primer sequences are listed in Table 1.

**Table 1:** qPCR primer sequences

	Forward primer (5′–3′)	Reverse primer (5′–3′)
GAPDH	TGTACCACCAACTGCTTAGC	GGCATGGACTGTGGTCATGAG
ANOS1	AACTCCAGCCAGACTGTGAC	GAGTGGGTCGTCGTCTTTGAA
MMP3	CACTCACAGACCTGACTCGG	AGTCAGGGGGAGGTCCATAG
MMP1	ACCTGGAAAAATACTACAACCTGAA	TTCAATCCTGTAGGTCAGATGTGTT
MT1F	AGTCTCTCCTCGGCTTGC	ACATCTGGGAGAAAGGTTGTC
COL10A1	TCCTTGAACTTGGTTCATGGAGT	ACTGTGTCTTGGTGTTGGGTAGTG
POSTN	TGCCCAGCAGTTTTGCCCAT	CGTTGCTCTCCAAACCTCTA
CXCL5	GAGAGCTGCGTTGCGTTTG	TTTCCTTGTTTCCACCGTCCA
CXCL6	ACGCTGAGAGTAAACCCCAA	CCAGACAAACTTGCTTCCCG
HAPLN1	CAGACCTCACTCTGGAAGATTATG	GGGAATACCAGACCTTGTAAGT
AIM2	CAGAAGGTAACAGAAAAGAAGA	ACAGTGTGAAGAATGTAAGTC
SCX	AGAACACCCAGCCCAAACAGAT	TCGCGGTCCTTGCTCAACTTT
MKI67	TGACCCTGATGAGAAAGCTCAA	CCCTGAGCAACACTGTCTTTT
BUB1	TGGGACTGTTGATGCTCCAAAC	GGAACTCACTGGTTTAGAAAGCCCAG
BMP2	CCCTACATGCTAGACCTGTATCG	TCCTCCGTGGGGATAGAAC
TGFBR1	GACAACGTCAGGTTCTGGCTCA	CCGCCACTTTCCTCTCCAAACT

### Statistical analysis

Data were presented as mean ± standard deviation (SD). One-way ANOVA with post hoc Tukey tests was performed using GraphPad Prism v8.4.2. *P*-values < 0.05 were considered statistically significant. Statistically significant differences were indicated as: *, *P* < 0.05; **, *P* < 0.01; and ***, *P* < 0.001.

## Results

### Characterization of tECM protein composition

**Fig. 1:**
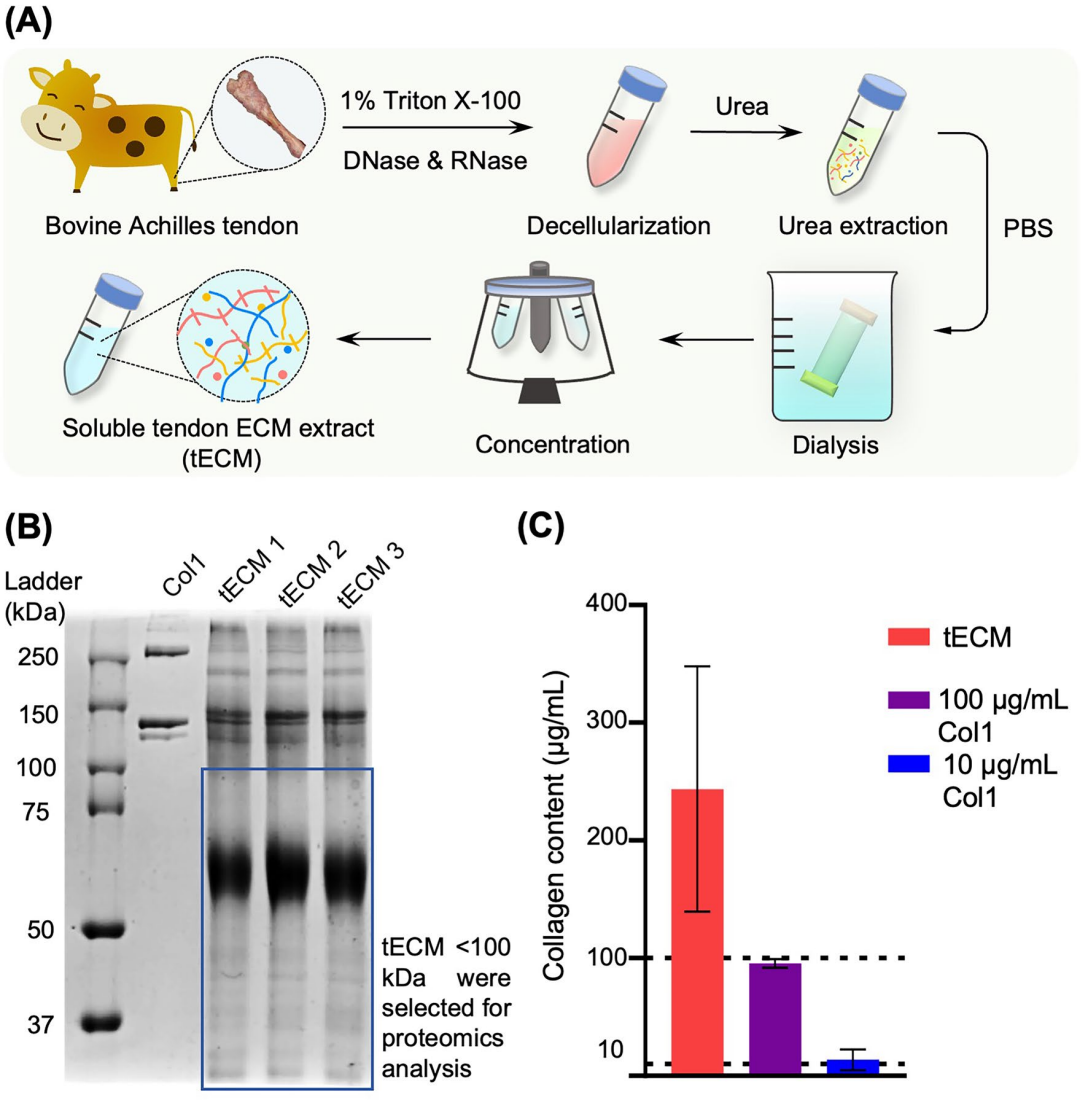
tECM extraction and characterization. **A** Schematic diagram of urea-based tECM extraction. **B** SDS-PAGE showed that tECM contains abundant low to medium molecular weight proteins that are absent in commercial collagen type I preparation (Col1). **C** Collagen content analysis (hydroxyproline assay calibrated with collagen type I standards) showed that the average collagen content was 246.32 μg/mL in a 1 mg/mL tECM solution

Urea extraction of tendon ECM is shown in Fig. 1A. SDS-PAGE analysis of individual batches of tECM extracts showed a consistent protein pattern, including collagen bands (*α*1 and *α*2 chains) and low to medium molecular weight proteins (< 100 kDa) (Fig. 1B). Hydroxyproline assay of the different tECM batches showed an average collagen content of 246.32 ± 106.70 μg/mg protein (mean ± SD) in the tECM solution (Fig. 1C).

**Fig. 2:**
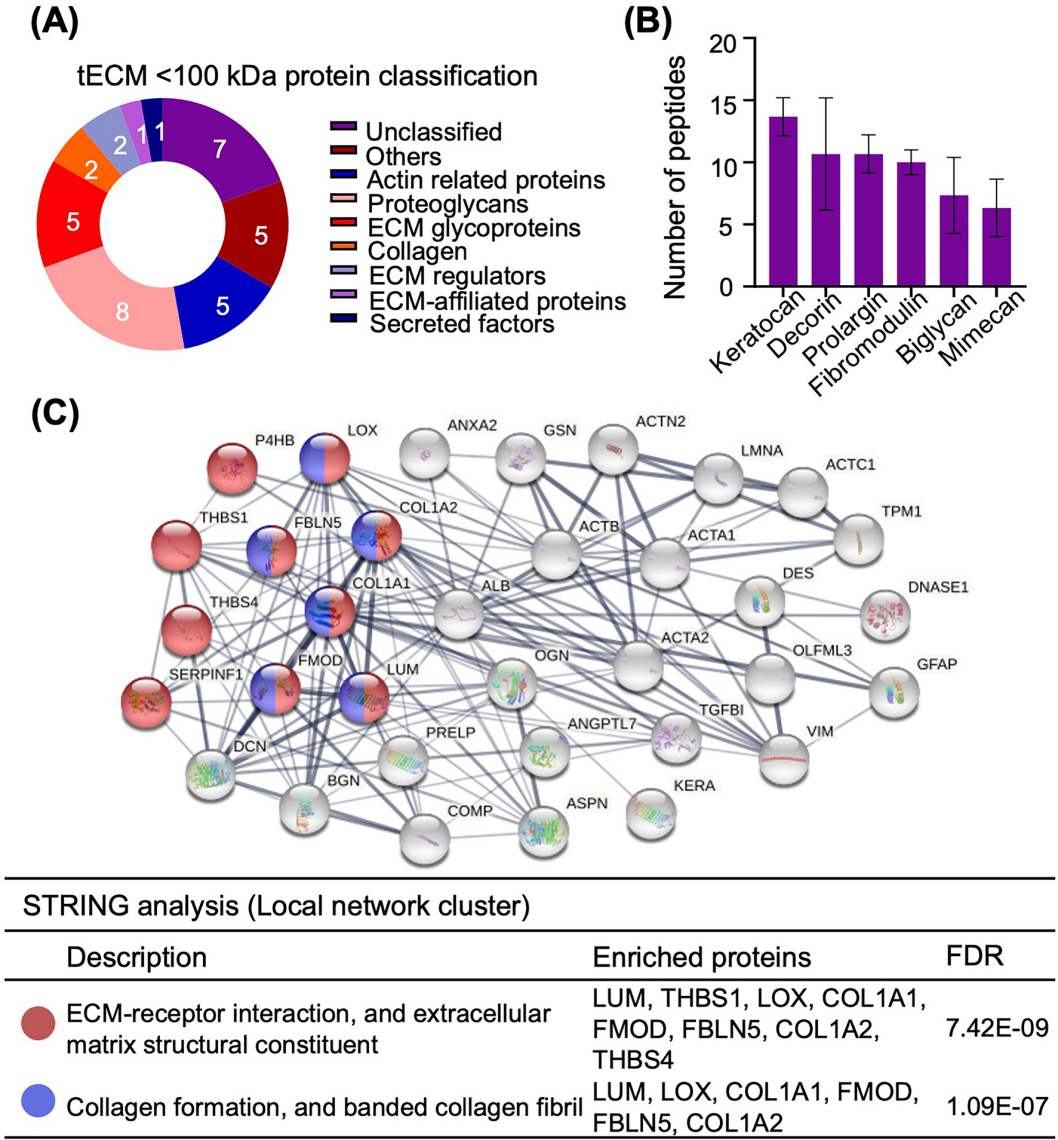
Protein composition analysis of tECM. **A** Protein bands < 100 kDa from three batches of tECM were extracted from SDS-PAGE gel, subjected to in-gel tryptic digestion and further analyzed by mass spectrometry. A total of 29 proteins were identified based on the Matrisome Project and PANTHER classification system. *n* = 3 isolates; mean ± SD. **B** Top identified ECM proteins in tECM (< 100 kDa). **C** Protein–protein interaction (PPI) based on STRING local network cluster analysis [confidence score threshold at 0.4 (medium)] highlights significant protein interaction networks. Proteins are represented as nodes of different colors

Compared to the extensive studies on the structural and biological roles of collagens in tendon, the functional aspects of the non-collagenous ECM components are less defined. To further identify low to medium molecular weight proteins in tECM, proteins < 100 kDa in molecular weight from three batches of tECM were extracted from the SDS-PAGE gel and subjected to in-gel tryptic digestion. Thirty-six proteins were identified as listed in Table 2. Based on ‘The Matrisome Project,’ which was used to define the putative ECM proteins in silico and proteomic approaches [17], 19 tECM proteins (< 100 kDa) were categorized as core matrisome and matrisome-associated proteins. These proteins included collagens (6%), proteoglycans (22%), glycoproteins (14%), secreted factors (3%), ECM-affiliated proteins (3%) and ECM regulators (6%) (Fig. 2A). The top 6 ECM proteins based on identified numbers of peptides (mean of three batches) are shown in Fig. 2B. Specifically, Keratocan (playing a pivotal role in collagen fibrillogenesis) [25], prolargin (anchoring basement membranes) [26] and decorin ("decorates" collagen type I, involved in cell differentiation and collagen fibrillogenesis) were the major proteoglycans that contribute to collagen binding and matrix structure maintenance (Fig. 2B) [13, 14]. The major glycoproteins identified in tECM extracts (< 100 kDa) were cartilage oligomeric matrix protein (COMP, thrombospondin 5), thrombospondin 1 (TSP 1) and TSP 4 (Table 2). The proteins from the TSP family can bind with different ECM proteins and help with ECM synthesis [13]. STRING analyses resulted in a dense network of proteins with two highly connected clusters centered around ECM-receptor interaction and collagen formation (Fig. 2C).

**Table 2:** Identified tECM proteins (< 100 kDa)

Protein name	Gene symbol	Category	Molecular weight (kDa)	Isoelectric point (pI)	Number of peptides	Sequence coverage (%)
Keratocan	KERA	Proteoglycans	40.4	6.8	14	36.53
Prolargin	PRELP	Proteoglycans	43.7	9.6	11	31.60
Decorin	DCN	Proteoglycans	39.9	8.7	11	30.07
Vimentin	VIM	Unclassified	53.7	5.0	10	24.60
Fibromodulin	FMOD	Proteoglycans	43.0	5.6	10	32.20
Actin, alpha cardiac muscle 1	ACTC1	Actin-related proteins	42.0	5.2	10	37.70
Actin, alpha skeletal muscle	ACTA1	Actin-related proteins	42.0	5.2	10	33.93
Actin, aortic smooth muscle	ACTA2	Actin-related proteins	42.0	5.2	9	29.20
Biglycan	BGN	Proteoglycans	41.5	6.8	7	23.30
Actin, cytoplasmic 1	ACTB	Actin-related proteins	41.7	5.3	7	23.83
Alpha-actinin-2	ACTN2	Unclassified	103.7	5.3	7	8.95
Mimecan	OGN	Proteoglycans	34.2	5.4	6	17.97
Prelamin-A/C	LMNA	Unclassified	74.2	6.7	6	9.60
Angiopoietin-related protein 7	ANGPTL7	Secreted Factors	39.4	7.6	5	17.27
Annexin A2	ANXA2	ECM-affiliated Proteins	38.6	6.5	4	16.03
Thrombospondin-4	THBS4	ECM Glycoproteins	105.9	4.4	4	5.87
Cartilage oligomeric matrix protein	COMP	ECM Glycoproteins	82.3	4.4	4	8.03
Transforming growth factor-beta-induced protein ig-h3	TGFBI	ECM Glycoproteins	74.4	6.8	4	8.83
Serum albumin	ALB	Others	69.2	5.8	3	12.60
Olfactomedin-like protein 3	OLFML3	Others	45.9	6.2	2	7.73
Lumican	LUM	Proteoglycans	38.7	5.9	2	8.65
Fibulin-5	FBLN5	ECM Glycoproteins	50.1	4.6	2	5.10
Collagen alpha-1(I) chain	COL1A1	Collagen	138.9	5.6	2	3.53
Protein-lysine 6-oxidase	LOX	ECM Regulators	29.1	6.0	2	13.30
Pigment epithelium-derived factor	SERPINF1	ECM Regulators	46.2	6.6	2	7.00
Tropomyosin 1	TPM1	Actin-related proteins	32.7	4.7	2	9.90
Desmin	DES	Unclassified	53.5	5.2	2	6.00
Deoxyribonuclease-1	DNASE1	Unclassified	31.3	5.3	2	11.40
Thrombospondin-1	THBS1	ECM Glycoproteins	129.5	4.7	1	1.37
Gelsolin	GSN	Others	80.7	5.5	1	1.50
Asporin	ASPN	Proteoglycans	42.1	9.2	1	5.70
Collagen alpha-2(I) chain	COL1A2	Collagen	129.0	9.2	1	1.60
Tumor necrosis factor alpha-induced protein 8-like protein 1	TNFAIP8L1	Unclassified	20.9	9.7	1	5.90
Tissue factor pathway inhibitor 2	TFPI2	Others	26.7	9.1	1	2.60
Protein disulfide-isomerase	P4HB	Others	56.8	4.8	1	3.10
Glial fibrillary acidic protein	GFAP	Unclassified	49.5	5.4	1	2.30

Our tECM proteomics results were further compared with current literature and are shown in Table 3. Using MS-based proteomics analysis, 19 ECM proteins (< 100 kDa) were identified in our work, while 34 to 85 ECM proteins were reported in the literature. Specifically, in our study, collagens (COL1A1 and COL1A2) and the non-collagenous proteins (DCN, FMOD, COMP and PRELP) were identified in tECM (< 100 kDa) [26–29]. Functional analysis of tECM (< 100 kDa) indicated that the principal GO processes were “extracellular matrix and collagen binding,” and two significant pathways, i.e., ECM-receptor interaction and focal adhesion, were identified by STRING analysis. Key ECM proteins and functional characterization identified in tECM were similar to other proteomic studies that characterized tendon ECM composition (Table 3) [26, 29].

**Table 3:** Comparison of tendon ECM proteomic study methodology and results

	Current study (tECM < 100 kDa)	Ref. [26]	Ref. [27]	Ref. [28]	Ref. [29]
Species	Bovines	Equine	Equine	Human	Canidae
Sample source	Achilles tendon	SDFT	SDFT	Patellar tendon	LDET
Sample preparation	3 M Urea	4 M GuHCL, 65 mM DTT	0.1% Rapigest	0.1% Rapigest in 50 mM ammonium bicarbonate	4 M GuHCL, 65 mM DTT
Protein digestion	In-gel trypsin digestion	In-solution trypsin digestion	In-solution trypsin digestion	In-solution trypsin digestion	In-solution trypsin digestion
Extracellular matrix (ECM) protein	Total: 19	Total: 36	Total: 34	Total: 63	Total: 85
Collagen	2	–	15	13	11
Proteoglycan	8	–	7	8	13
Glycoprotein	5	–	8	24	30
Main identified Collagen	COL1A1, COL1A2	COL1A1, COL1A2, COL3A1, COL4A1	COL1A1, COL1A2, COL12A1	COL1A1, COL1A2, COL3A1, COL12A1	COL1A1, COL1A2, COL6A1, COL6A2, COL12A1
Main identified non-collagen ECM protein	KERA, DCN, BGN, FMOD, PRELP, OGN	DCN, FMOD, COMP, THBS4, PRELP	FN, THBS4, FMOD, COMP, CILP1, THBS1	FMOD, FN1, PRELP, COMP	DCN, BGN, LUM, TNC
Identified protein-enriched GO functional annotation	Extracellular matrix; Collagen binding	Intermediate filament; Extracellular matrix	Organization of collagen fibrils and filaments	–	ECM organization; Wound healing; Collagen fibril organization
Identified protein-enriched pathway	ECM-receptor interaction; Focal adhesion	ECM-receptor interaction; Focal adhesion	–	–	–

Taken together, the tECM protein composition data showed that our urea-based tECM preparation represents an effective and highly reproducible approach of extracting bioactive ECM components from tendon. tECM contains multiple key tendon ECM proteins, as well as components involved in GO functions (ECM-receptor interaction and collagen formation) and signaling pathways (ECM-receptor interaction and focal adhesion), which were similarly reported in the literature.

### Comparison between pro-tenogenic activity of tECM and Col1 on hASCs

**Fig. 3:**
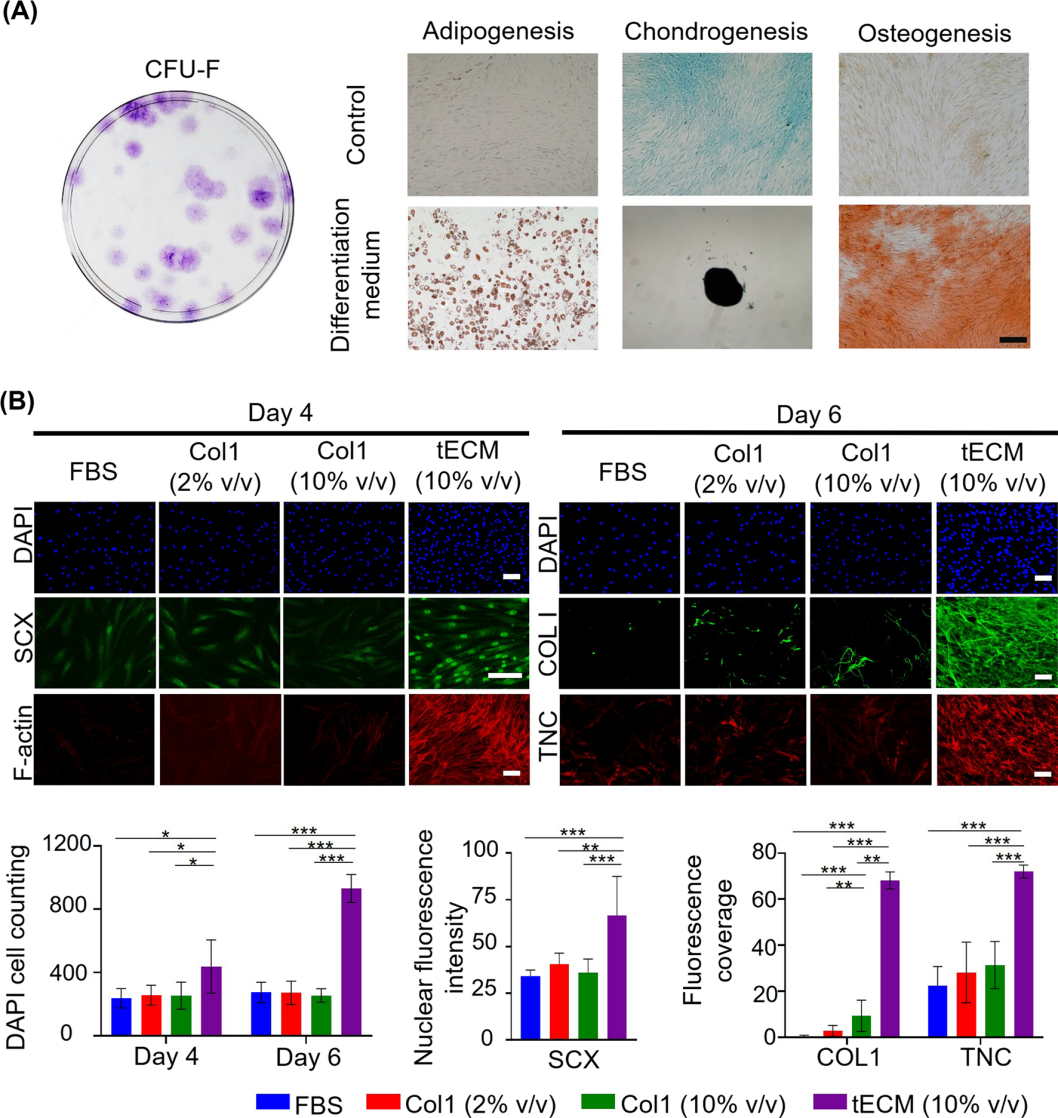
hASC characterization and pro-tenogenic effect of tECM on hASCs. **A** hASCs were characterized by CFU-F assay and tri-lineage differentiation assay (adipogenesis: Oil red O staining; chondrogenesis: Alcian Blue staining; and osteogenesis: Alizarin red staining). **B** hASCs were cultured with FBS, Col1 (2% v/v or 10% v/v) or tECM (10% v/v) for indicated duration. tECM group showed significantly enhanced cell proliferation (DAPI) and staining intensity for COL1, SCX, TNC, and F-actin. Top: fluorescence images; bottom: quantitation of stained cell number (DAPI) and staining intensity (SCX, COL1, and TNC). *n* = 3 isolates; $$*$$, *P* < .05, $$**$$, *P* < .01; $$***$$, *P* < .001, scale bar: 200 µm

Protein composition analysis showed that tECM contained multiple key tendon ECM components in addition to collagens, suggesting that tECM could exhibit superior pro-tenogenic bioactivity on hASCs compared to Col1 solution alone. This hypothesis was tested using hASCs sorted by flow cytometry for mesenchymal stem cell characteristics (positive markers: CD44, CD73, CD90 and CD105; negative markers: CD11b, CD19, CD34, CD45 and HLA-DR) (data not shown). The self-renewal (CFU-F assay) and multi-differentiation potential (adipogenesis, osteogenesis and chondrogenesis) of the sorted hASCs were validated (Fig. 3A). hASCs, Passages 4–7, were used in subsequent studies [16]. Hydroxyproline assay was performed to quantify the collagen content of tECM, revealing approximately 0.2 mg/mL collagen in 1 mg/mL tECM. Therefore, tenogenic differentiation of hASCs cultured in tECM (10% v/v), Col1 (2% v/v or 10% v/v) and control basal medium (FBS) for 4 or 6 days were assessed by immunofluorescence staining of tenogenesis-associated markers (SCX, COL1 and TNC), F-actin staining and DAPI nuclear staining (Fig. 3B). DAPI- based cell counting showed significantly increased cell proliferation in the tECM group compared to the other three groups. More pronounced immunostaining of SCX, COL1 and TNC, and F-actin staining was also observed in the tECM-treated group compared to the other three groups. Specifically, enhanced nuclear staining of SCX, a tendon-specific transcription factor, as well as dense extracellular collagen and TNC fibril network were found in tECM-treated, but not Col1-treated hASCs. Normalized, semi-quantitative analyses of immunofluorescence staining intensity at culture days 4 and 6 validated a trend of more intense staining of tenogenesis-associated markers in the tECM group compared to the FBS and Col1 (2% v/v or 10% v/v) groups (Fig. 3B).

Taken together, these findings showed that treatment with tECM enhanced hASC proliferation and tenogenic differentiation compared to Col1 treatment. Thus, while Col1 is the major ECM component of tendon, other non-collagenous tendon ECM components are likely to contribute to its pro-tenogenesis bioactivity.

### Transcriptomic landscape of tECM-driven pro-tenogenic differentiation of hASCs

**Fig. 4:**
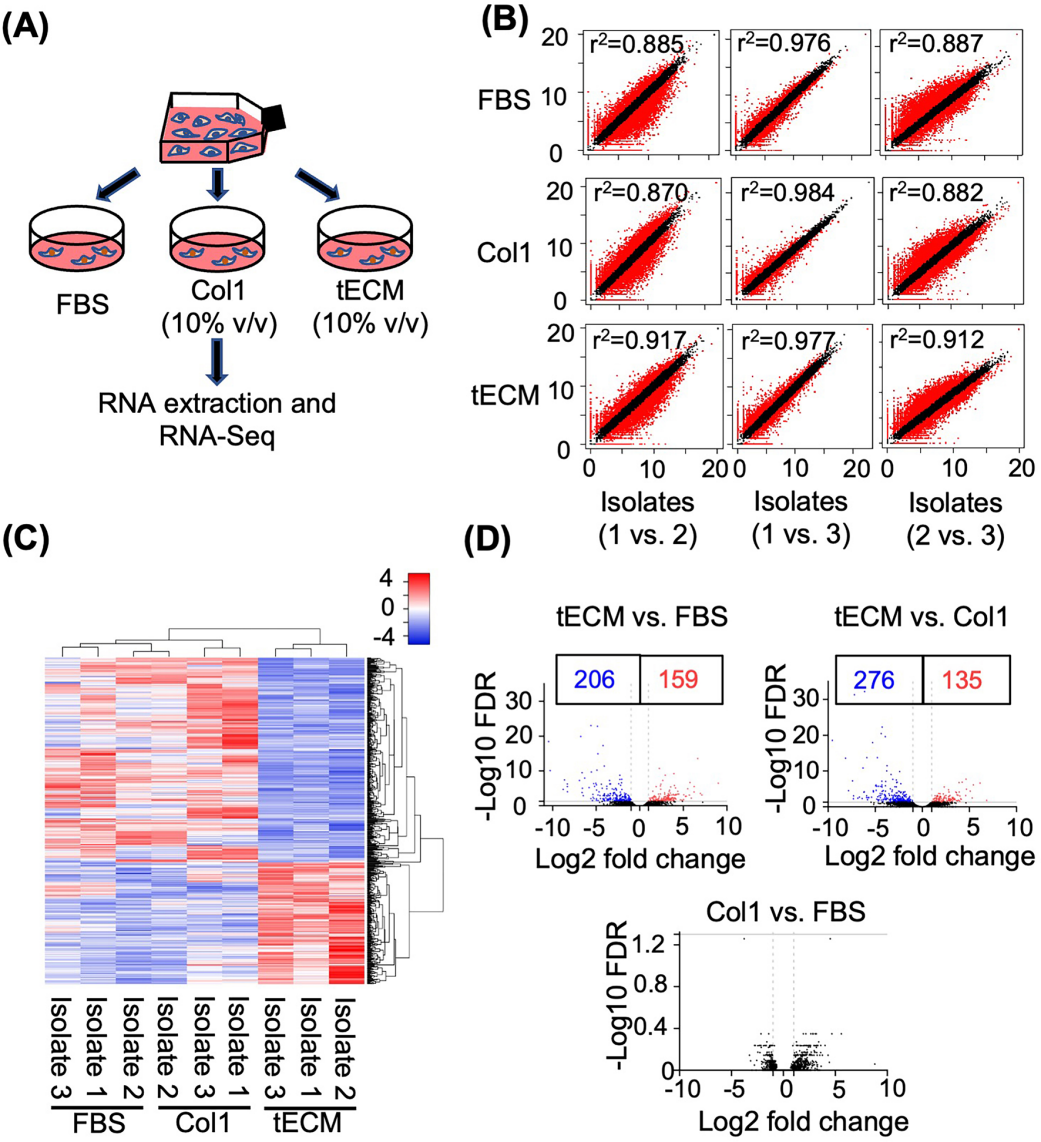
RNA-Seq transcriptome analysis. **A** hASCs were cultured with FBS, Col1 or tECM for 6 days and total cellular RNA was harvested for RNA-Seq analysis. **B** Scatter plots indicate the correlation (*r*^2^) between the isolates under different culture conditions. **C** Heat map of transcriptome analysis for 584 differentially expressed genes (DEGs) from three culture groups. **D** Volcano plots of transcriptome: red spots, upregulated genes; blue spots, downregulated genes; and black spots, unchanged genes. DEGs are designated for those exhibiting > twofold changes and FDR < 0.05

To investigate the molecular pathways and mechanisms underlying the tECM-driven pro-tenogenic differentiation of hASCs, total cellular RNA was extracted from hASCs cultured under three different conditions (FBS, tECM (10% v/v) or Col1 (10% v/v)) for RNA-Seq analysis (Fig. 4A). RNA-Seq analysis was performed using three independent isolates (biological replicates) of each culture condition and sequenced to 61, 482, 962–88, 727, 892 raw reads per library. The similarity of expression profiles was determined by Pearson correlation coefficient (PCC) analysis, which showed a high correlation (R^2^ ranging from 0.84 to 0.98) in gene expression profiles among 3 isolates as visualized using scatter plotting (Fig. 4B). Taken together, these data show a high degree of similarity among different biological replicates for each culture condition. To analyze and compare gene expression among the three culture groups (tECM versus FBS, Col1 versus FBS, tECM versus Col1), an R package—DESeq was used as described previously [30, 31]. A total of 584 genes were differentially expressed (FDR < 0.05) with an absolute fold change of 2 or greater between comparisons (Fig. 4C). In particular, 365 DEGs were found in the tECM group compared to the FBS group, with 159 upregulated genes and 206 downregulated genes as shown in the volcano plot. In addition, 411 genes were differentially expressed in the tECM group compared with the Col1 group, with 135 upregulated genes and 276 downregulated genes. On the other hand, no DEG was found between Col1 and FBS groups (Fig. 4D).

**Fig. 5:**
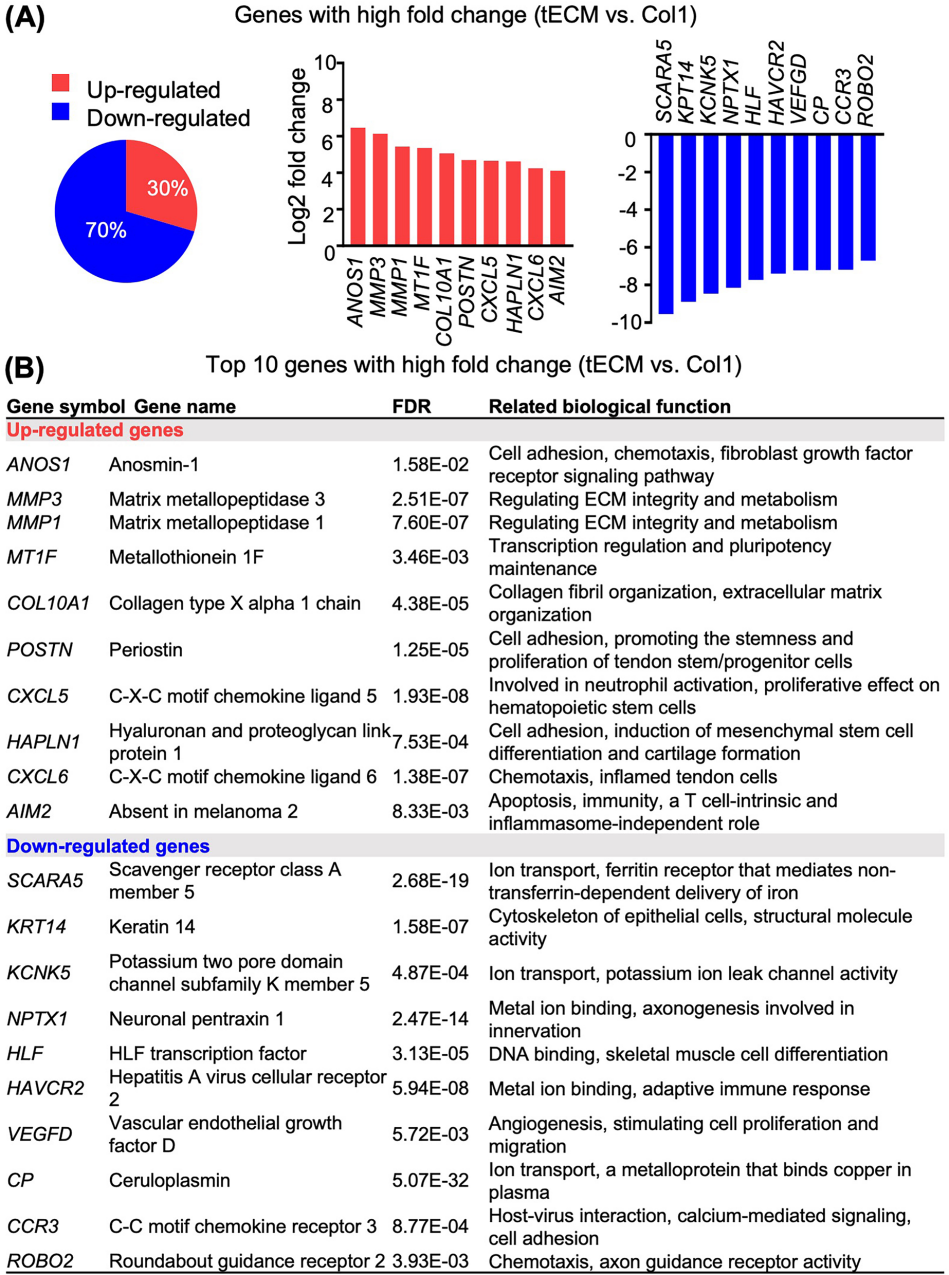
Comparison of gene expression between tECM and Col1 treatment groups. **A** Percentage and fold change expression of (red) up- or (blue) downregulated genes (> twofold changes). **B** Top 10 up- and downregulated genes and their related biological functions. All fold changes are log 2 base transformed

Heat map and volcano plot highlighted that the tECM group exhibited distinct gene expression profiles compared with the other two groups, while the difference in gene expression between Col1 and FBS groups was minor (Fig. 4C, D). Specifically, more downregulated genes (around 70%) were shown in the tECM group compared with Col1 group (Fig. 5A). The top ten upregulated and downregulated genes as well as their related biological functions, which include regulation of cell fate, stemness, proliferation and chemotaxis as well as ECM metabolism, are shown in Fig. 5A and B.

### Functional annotation and pathway analysis between the tECM and Col1 treatment groups

To explore and functionally classify differentially regulated genes (> twofold changes) between the tECM and Col1 groups, GO enrichment, KEGG pathway and GSEA analyses were performed.

**Fig. 6:**
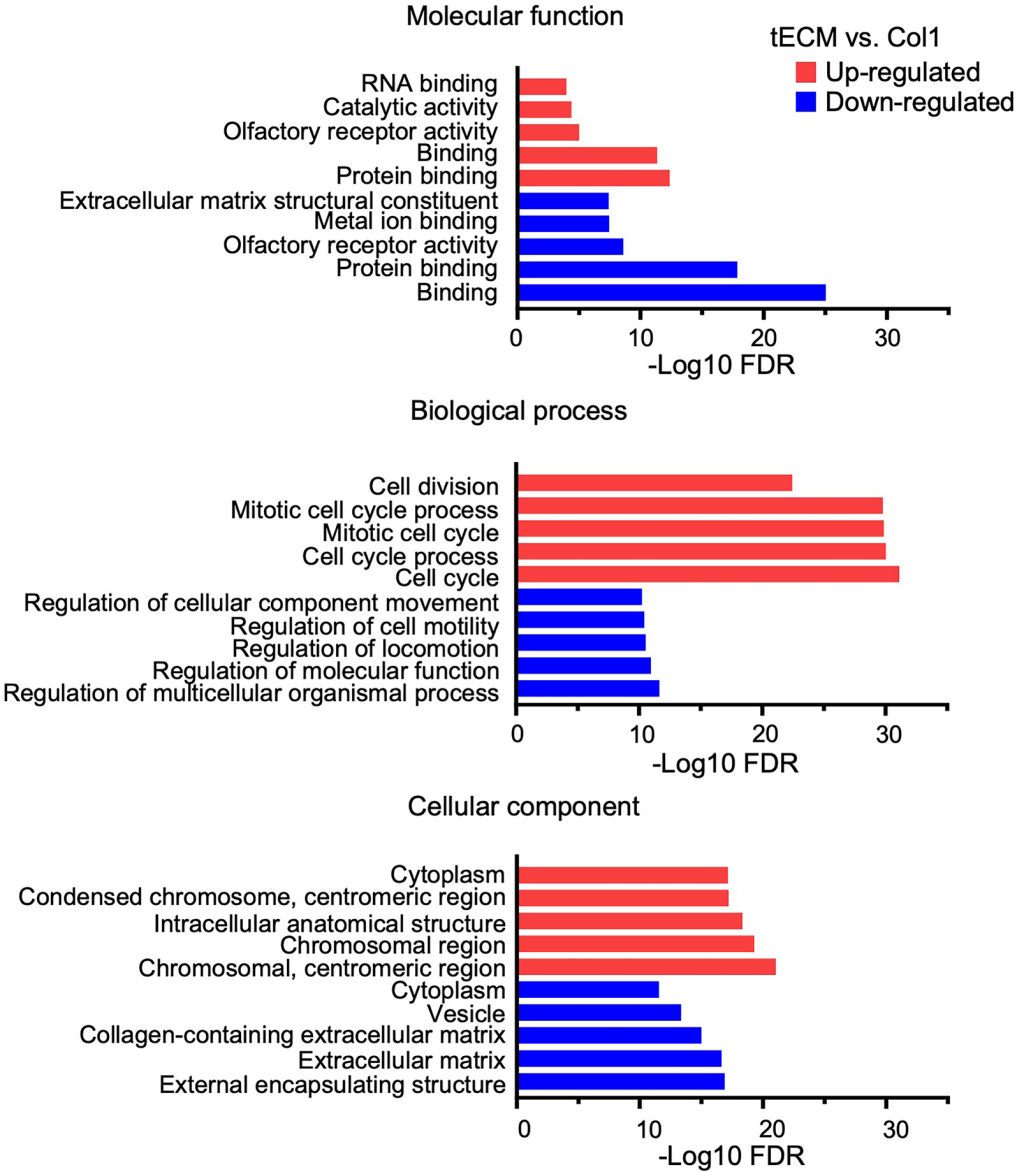
GO enrichment analysis of differential gene expression between tECM and Col1 treatment groups. Top 5 upregulated (red) and top 5 downregulated (blue) GO terms are shown, including molecular functions, biological processes and cellular components. Genes exhibiting > twofold changes between the tECM and Col1 groups are selected, and all FDR values are log 10 base negative

tECM and Col1 groups generally exhibited differential enrichment in GO terms. The upregulated genes in tECM group compared to Col1 group were found to be enriched in: (1) molecular functions, e.g., “RNA binding” and “catalytic activity”; (2) biological processes associated with cell behaviors, e.g., “cell division” and “cell cycle process”; and (3) cellular components associated with “chromosomal region,” “condensed chromosome” and “centromeric region.” Relative to tECM group, the Col1 group showed up-regulation of ECM-associated processes, such as “ECM structural constituents,” “metal ion binding” and “collagen-containing ECM,” suggesting involvement in extracellular structural organization (Fig. 6). The similarity was also found between tECM and Col1 groups for GO function associated with “protein binding/glycoprotein binding” [32], which is important for ECM synthesis and assembling.

**Fig. 7:**
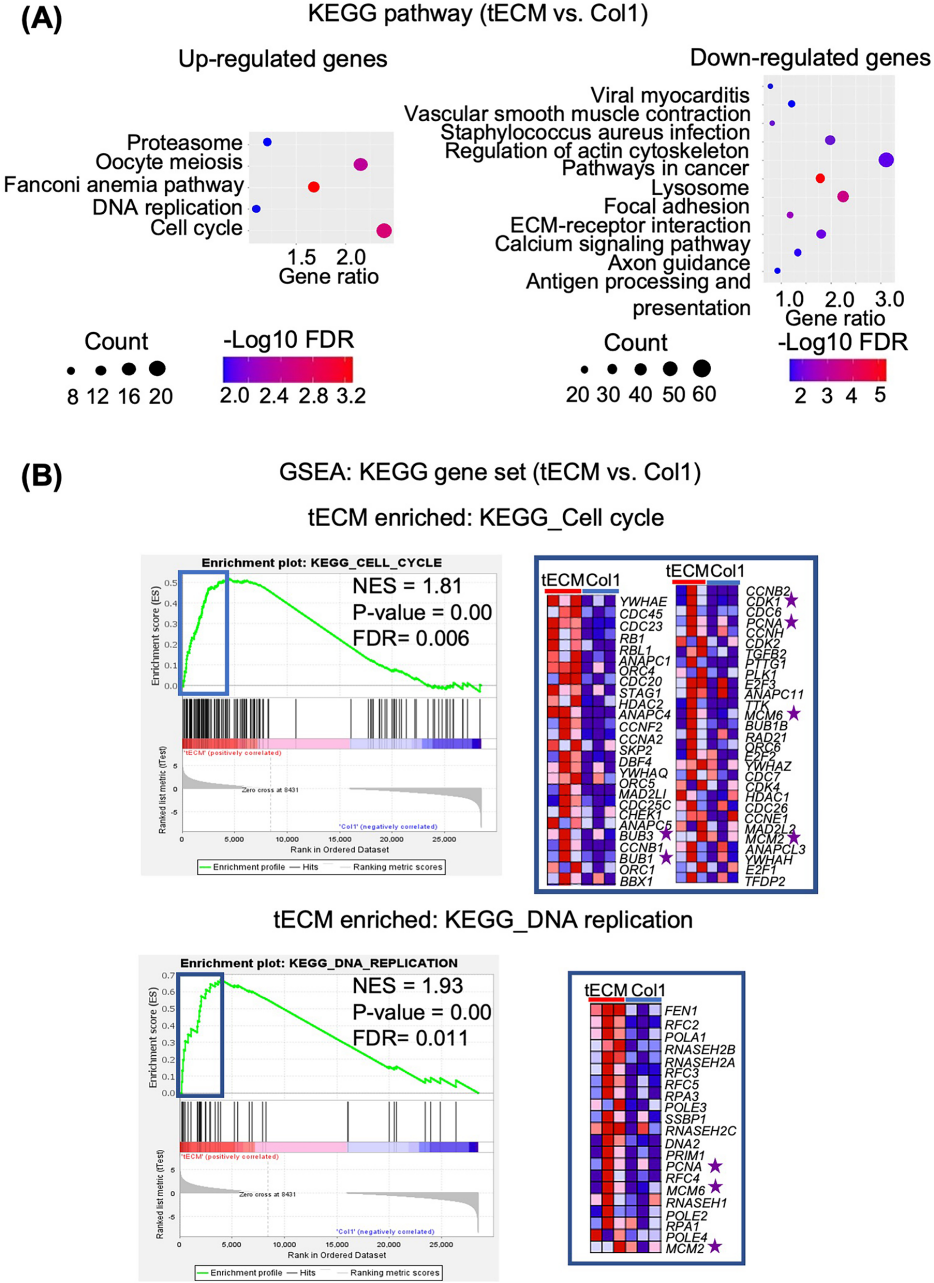
Pathway analysis of differential gene expression between tECM and Col1 treatment groups. **A** KEGG pathway analysis. Vertical—KEGG pathway, and horizontal—gene ratio, in the advanced bubble charts. The size and color of the bubbles represent the count and FDR value (log 10 transformed), respectively, of differential genes enriched in each pathway.** B** GSEA showing that the tECM group was significantly enriched in pathways associated with “cell cycle” (FDR = 0.006) and “DNA replication” (FDR = 0.011), as well as genes included for cell proliferation, such as PCNA and MCM. Relative levels of gene expression (red, high; blue, low) of the core enrichment genes are shown in the heat map

Additionally, KEGG pathway analysis was performed to compare signaling network for genes with > twofold changes between the tECM and Col1 groups. The results showed that different signaling pathways were activated in hASCs between tECM and Col1 treatments. tECM group activated more cell activity-related pathways, such as “cell cycle progression and proteasome” pathways, which are involved in many essential biological processes, e.g., cell cycle and DNA replication. Col1 treatment activated more ECM-related signaling pathways, such as “focal adhesion and ECM-receptor interaction,” which play essential roles in cell-ECM interaction and the maintenance of cell/tissue structure and function (Fig. 7A).

GSEA is designed to detect modest but coordinated changes in the expression of functionally related gene groups [24, 33]. This analysis was performed on all expressed genes, thus addressing some inherent limitations of DEG-centric analyses [33]. GSEA was performed based on KEGG pathway gene sets comparing the tECM and Col1 groups (NES > 1, *P* < 0.05, and FDR < 0.25) (Table 4). Similar to the KEGG pathway results, the tECM group was significantly enriched in pathways associated with molecular activities, e.g., “cell cycle” (FDR = 0.006) and “DNA replication” (FDR = 0.011) (Fig. 7B). Specifically, the core enrichment genes of tECM group were found to be closely related to cell proliferation. For example, genes such as proliferating cell nuclear antigen (*PCNA*) and minichromosome maintenance (*MCM*), well-established markers for cell proliferation [34], were highly enriched in the KEGG cell cycle gene sets in the tECM group compared to the Col1 group (Fig. 7B).

**Table 4:** GSEA of KEGG pathway gene sets enriched in tECM and Col1 groups

KEGG pathway name	Gene set size	ES	NES	*P*-value	FDR
*KEGG gene sets enriched in tECM group*					
Oxidative phosphorylation	131	0.67	2.37	0.00E+00	0.00E+00
Parkinson's disease	127	0.66	2.36	0.00E+00	0.00E+00
Proteasome	46	0.78	2.35	0.00E+00	0.00E+00
Alzheimer's disease	165	0.54	2.00	0.00E+00	9.26E−04
DNA replication	36	0.67	1.93	0.00E+00	2.69E−03
Huntington's disease	180	0.52	1.92	0.00E+00	2.49E−03
Protein export	24	0.71	1.90	0.00E+00	2.84E−03
RNA degradation	59	0.57	1.82	0.00E+00	6.94E−03
Cell cycle	123	0.52	1.81	0.00E+00	6.30E−03
Pyrimidine metabolism	98	0.53	1.81	0.00E+00	6.18E−03
Spliceosome	127	0.50	1.78	0.00E+00	7.30E−03
RNA polymerase	29	0.62	1.71	0.00E+00	1.68E−02
Aminoacyl tRNA biosynthesis	40	0.56	1.63	0.00E+00	3.13E−02
Ribosome	88	0.48	1.63	8.58E−03	2.96E−02
N glycan biosynthesis	46	0.50	1.50	9.93E−03	7.35E−02
Steroid biosynthesis	17	0.69	1.65	1.07E−02	2.75E−02
Oocyte meiosis	112	0.38	1.31	1.11E−02	2.01E−01
Ubiquitin-mediated proteolysis	134	0.36	1.30	2.02E−02	1.99E−01
Riboflavin metabolism	16	0.65	1.53	2.55E−02	6.55E−02
Nucleotide excision repair	44	0.45	1.34	4.19E−02	1.82E−01
*KEGG gene sets enriched in Col1 group*					
Calcium signaling pathway	177	− 0.54	− 1.71	0.00E+00	9.22E−02
Complement and coagulation cascades	69	− 0.57	− 1.62	1.39E−03	1.64E−01
Cell adhesion molecules cams	133	− 0.51	− 1.58	0.00E+00	2.01E−01
Endocytosis	181	− 0.49	− 1.56	0.00E+00	1.94E−01

*ES* enrichment score, *NES* normalized enrichment scored, *FDR* false discovery rate

Taken together, the results from the GO enrichment, KEGG pathway and GSEA analyses indicated that Col1-treated hASCs predominantly exhibited ECM-associated processes, while tECM-treated hASCs expressed a mixture of ECM- and proliferation-associated response. These findings provide partial explanation of the enhanced proliferation and pro-tenogenesis effects of tECM treatment on hASCs.

### Assessment of tenogenesis-associated genes between the tECM and Col1 treatment groups

To further assess the molecular mechanisms of the pro-tenogenesis activity of tECM on hASCs, gene expression between tECM and Col1 groups was directly compared in terms of proliferation and tenogenesis-associated genes.

**Fig. 8:**
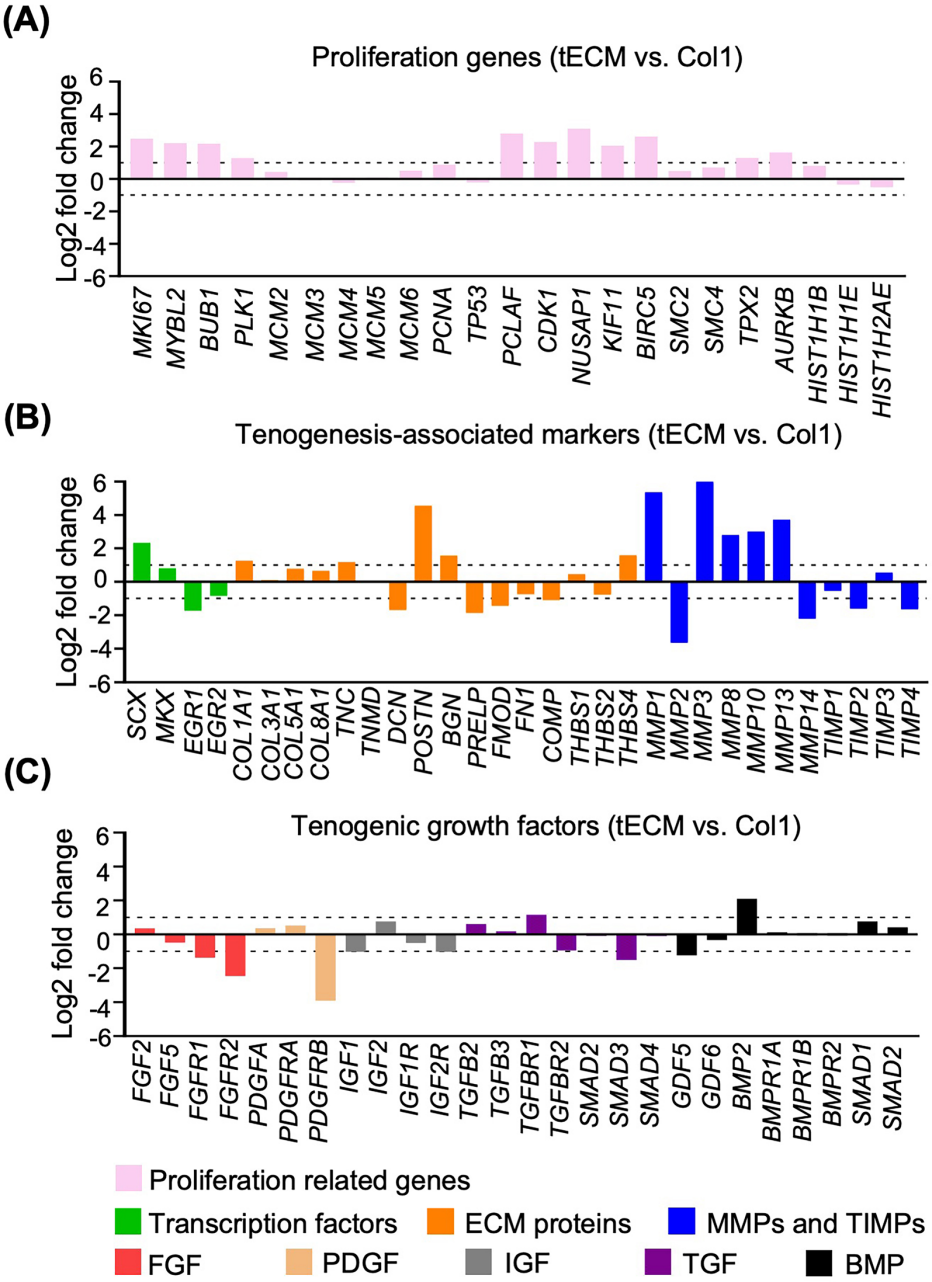
Comparison of proliferation and tenogenic gene expression between tECM and Col1 treatment groups. **A** Proliferation-related genes.** B** Tenogenesis-associated genes. **C** Tenogenic growth factors. Genes exhibiting > twofold changes between tECM and Col1 groups are analyzed. All fold changes are log 2 base transformed

A list of proliferation genes was selected, such as marker of proliferation Ki-67 (*MKI67*), *PCNA* and *MCM*, which are established cell proliferation markers [34]. Additionally, another set of genes directly associated with cell proliferation, mitotic process and cell division [e.g., myeloblastosis proto-oncogene like 2 (*MYBL2*), budding uninhibited by benzimidazoles 1 (*BUB1*), polo-like kinase 1 (*PLK1*), cyclin-dependent kinase 1 (*CDK1*) and kinesin family member 11 (*KIF11*)] were also included [34–36]]. Based on our data, more abundant proliferation genes were upregulated in the tECM compared to Col1 groups, such as *MKI67*, *MYBL2*, *BUB1*, *PLK1*, *CDK1* and *KIF11* (Fig. 8A).

For expression of tenogenesis-associated genes, our analysis focused on tenogenic transcription factors, ECM and growth factor signaling molecules (Fig. 8B, C). For transcription factors, *SCX*, mohawk (*MKX*) and early growth response transcription factors (*EGR1 and EGR2*) play critical roles in tendon lineage differentiation and are required for the regulation of collagen fibrils [37, 38]. Our data showed that the tECM group exhibited higher expression of *SCX* and *MKX*, but lower expression of *EGR1* and *EGR2* compared with Col1 treatment group. For tendon ECM-related genes, in addition to collagens, small leucine-rich proteoglycans (SLRPs), members of the leucine-rich repeat protein family, members of the TSPs family, matrix metalloproteinases (*MMPs*) and tissue inhibitor of metalloproteinases (*TIMPs*) have been shown to be important regulators for ECM synthesis and remodeling [14]. In our transcriptome dataset, the tECM group showed higher expression of collagen type I alpha 1 chain (*COL1A1*), *TNC*, biglycan (*BGN*), periostin (*POSTN*) and thrombospondins 4 (*THBS4*), whereas the Col1 group showed higher expression in decorin (*DCN*), prolargin (*PRELP*), fibromodulin (*FMOD*), fibronectin (*FN1*) and *COMP*. Importantly, most *MMPs* were upregulated and *TIMPs* were downregulated in tECM group compared to Col1 group, suggesting active ECM remodeling (Fig. 8B). For growth factors, the tECM group showed downregulated expression of some well-characterized tenogenic signaling growth factors, such as FGF, PDGF and IGF, with upregulated expression of TGF-*β* receptor 1 (*TGFBR1*) and *BMP2* (Fig. 8C). Interestingly, the expression of tenomodulin (*TNMD*), another tendon-specific membrane glycoprotein, could not be detected in either tECM or Col1 treated groups.

**Fig. 9:**
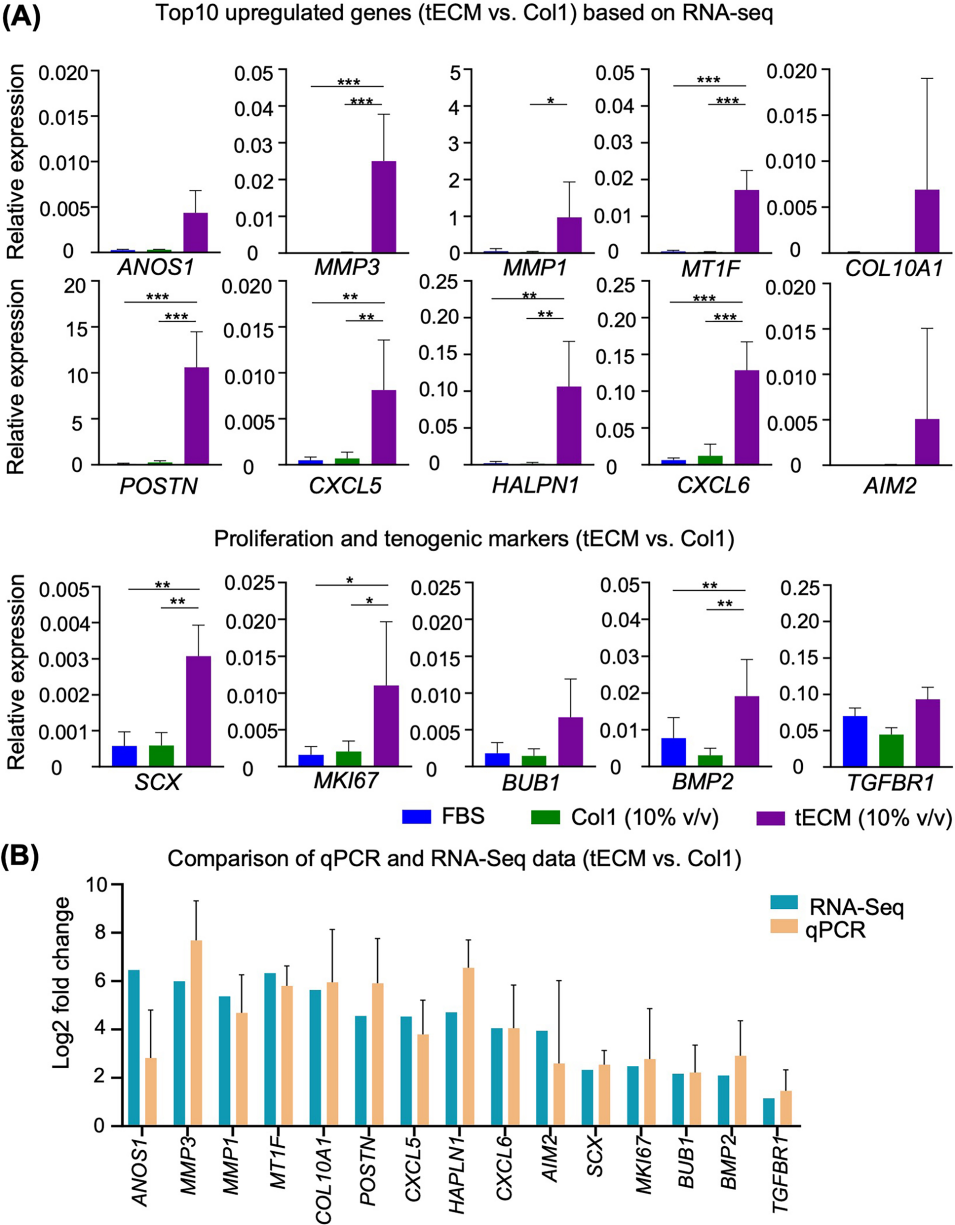
qPCR validation of RNA-seq results. **A** qPCR analysis of expression of genes identified by RNA-seq experiments to be positively differentially expressed in the tECM group, including the top 10 upregulated genes as well as proliferation-related and tenogenic-associated genes. **B** Comparison of gene expression levels between RNA-seq and qPCR (*n* = 5 isolates; *, *P* < .05; **, *P* < .01; ***, *P* < .001)

Additionally, in order to validate the gene expression profiles obtained from RNA-seq analysis, the expression of the top 10 upregulated genes, as well as selected proliferation and tenogenic markers, was further assayed by qPCR. Comparative qPCR analysis showed that, compared to Col1 (10% v/v) group, tECM-treated hASCs exhibited significantly increased expression of genes, including most of the top 10 upregulated genes identified in RNA-seq, proliferation gene *MKI67* and tenogenesis-associated genes (*SCX* and *BMP2*) at days 6. In comparison, no significant difference was observed in expression of these genes between the FBS and Col1 (10% v/v) groups (Fig. 9A, B). These qPCR results thus validated the expression profile obtained using RNA-seq.

Taken together, these results showed that tECM treatment induced in hASCs a gene expression profile distinct from that in the Col1 treated group. In particular, the tECM group showed higher expression of cell cycle- and tenogenesis-associated genes, likely contributing to the augmented cell proliferation and the pro-tenogenic effect of tECM on hASCs.

## Discussion

We have previously developed a urea-based, non-proteolytic method to extract bovine tendon ECM (tECM), which exhibited strong proliferation and tenogenic differentiation effects on hASCs [15, 16] and has potential application for tendon repair and regeneration. In this study, we have further characterized the protein composition of tECM, as well as its pro-tenogenic bioactivity and the corresponding transcriptome profile of hASCs cultivated in tECM, in comparison with those exposed to Col1. The results showed that: (1) the urea-extracted tECM contained around both collagen (20% w/w) and other non-collagenous tendon ECM components. Compared to the current MS-based proteomic studies on tendon ECM composition, our urea-extracted tECM contained multiple key tendon ECM proteins, as well as those that are involved in similar GO functions (ECM-receptor interaction and collagen formation) and signaling pathways (ECM-receptor interaction and focal adhesion); (2) compared to Col1 treatment, tECM supplementation enhanced hASC proliferation and promoted tenogenic differentiation as well as induced distinct gene expression profiles.

Optimal ECM extraction for biomedical applications needs to meet two key criteria, i.e., high efficiency of extraction and robust maintenance of the bioactivity of the ECM components, which is largely dependent on extraction protocols [39]. Acid-pepsin digestion has been the conventional method to extract tissue ECM for clinical applications [10]. While acid-pepsin is mostly efficient for the isolation of collagen, as most proteins are susceptible to pepsin-mediated proteolytic digestion, except the triple helical collagens; thus, as expected and reported by us and others [10, 40], pepsin solubilized ECM products contained mostly collagens and other structural ECM proteins, and few non-collagenous components [10, 40]. Moreover, pepsin digestion can alter the bioactivity of essential bioactive molecules, e.g., the potency of growth factors can be significantly reduced upon pepsin treatment [41–43]. In this work, we have chosen urea as a reagent to extract tendon ECM fractions, since urea denatures via disruption of hydrogen bonding that mediates lipid–lipid, lipid–protein and protein–protein interactions to solubilize tissue ECM in a manner that allows renaturation [44]. In addition, Naba et al. revealed that the abundance of fibrillar collagens could lower the extraction efficacy of other bioactive ECM-associated components. Thus, using urea as an extraction reagent can help increase the relative content of non-collagenous components since crosslinked fibrillar collagen remains largely insoluble in urea [39]. Furthermore, other studies showed that urea-extractable collagen resulted in different fibroblast behavior, such as higher cell motility, suggesting the suitability of urea-extracted ECM for biotechnological applications and tissue engineering [45].

To characterize urea-extracted tendon ECM, we investigated tECM protein patterns via SDS-PAGE, collagen content via hydroxyproline assay and protein composition via MS-based analysis and comparison with the current literature. Our results showed that urea-extracted tECM yielded consistent protein patterns (as detected by SDS-PAGE), with collagens (20% w/w) and at least 17 non-collagenous proteins (< 100 kDa) based on MS analysis. These proteins include a number of SLRPs, such as keratocan, fibromodulin and decorin, which are known to be involved in collagen fibril maturation and collagen fibrillogenesis [13, 14, 25]. As for glycoproteins, the top identified components are those of the TSP protein family, which contribute to regulating diverse cellular processes such as inflammation, cell migration and cell proliferation [13]. Other tendon ECM components previously identified in various MS studies, including decorin, biglycan, lumican and COMP, are also included in our tECM preparation [29, 46]. Additionally, functional analysis of tECM (< 100 kDa) using MS-based proteomics indicated similar principal GO processes and signaling pathways compared to the existing literature (Table 3) [26, 29]. Thus, our work suggests that our urea-based tECM preparation represents an efficient and highly reproducible approach of extracting these bioactive ECM components from tendon.

Meanwhile, we also noted that we had relatively low protein coverage (*n* = 36 proteins) identified by proteomics analysis, relative to the data reported for other tendon extracts (e.g., *n* = 215 [29] and *n* = 92 [27]). For example, Kharaz et al*.* [29] used GuHCL for tendon protein extraction (long digital extensor tendons) and identified around 215 proteins including 85 (around 40%) ECM proteins. Thorpe et al*.* used 0.1% Rapigest (a surfactant which offers a simple detergent-based extraction of tendon tissue) to extract proteins from the superficial digital flexor tendon and identified around 92 proteins with 34 (around 37%) ECM proteins [27, 47]. The discrepancy in comparison might be derived from several factors: (1) Our current study only focused on tECM proteins with molecular weight < 100 kDa, using an in-gel digestion protein extraction kit. This will eliminate ECM proteins with molecular weights > 100 kDa, such as collagens, fibronectin, fibrillin and other large proteoglycans (versican and aggrecan). (2) Compared to urea, which is a relatively mild, non-chaotropic reagent for ECM extraction, the use of GuHCL, a strong chaotropic denaturing reagent, could extract a larger number of intracellular and ECM proteins, while RapiGest extraction could result in an increased amount of identified collagens [47]. (3) In-gel trypsin digestion was used for protein sample preparation in our study. Sample loss was often more severe for gel-based method because extraction of peptides from a gel was inherently less efficient [48]. However, it is noteworthy that although GuHCL or RapiGest may be more efficient and extensive in protein extraction, urea is a milder denaturant and is thus expected to better maintain ECM bioactivity, which is important for the use of the extracted ECM for biomedical applications.

To gain insight into the mechanism of tECM bioactivity on hASCs, transcriptome expression profiles of hASCs cultivated in tECM or Col1 containing medium were compared by RNA-Seq and bioinformatics analysis. As shown in Fig. 4, tECM treatment triggered distinct hASC gene expression profiles compared to those of FBS and Col1 treatments, whereas gene expression between Col1 and FBS groups was relatively similar. GO enrichment and KEGG pathway analyses indicated different molecular mechanisms that the tECM group regulated cell proliferation, cell cycle and DNA replication pathways, while the Col1 group enhanced extracellular structure organization. Sun et al. performed single-cell RNA-Seq on human primary Wharton’s Jelly-derived MSCs (WJMSC) for studying functional characteristics associated with cell proliferation, development and inflammation response. Their work showed that upregulated genes in the subpopulations of WJMSCs, which possessed a higher proliferative capacity, were significantly enriched in the DNA replication pathway and cell cycle process [49]. Chen et al. characterized the tendon stem/progenitor cells (TSPC) from postnatal rat Achilles tendon tissue at different stages of development by microarray analysis. Their data suggested that TSPCs-7d (TSPCs isolated at postnatal day 7) had significantly higher proliferation ability, characterized by upregulated genes enriched in the GO terms related to mitosis, cell division, cell cycle and DNA polymerase-related regulation [50]. Therefore, the identification of cell cycle process and signaling pathway via DEGs, GO and KEGG pathway analyses upon tECM treatment suggests that these pathways are involved in the bioactivity of tECM on hASC behaviors, including proliferation and lineage-specific differentiation.

Proliferation and tenogenesis-associated gene expression, including tenogenic transcription factors, tendon ECM and tenogenic growth factor signaling molecules, were compared between tECM and Col1 groups. Although expression of *SCX*, *MKX*, *COL1A1*, *TNC* and *BGN* was upregulated in the tECM group, Col1 treatment also induced higher expression of some tenogenesis-associated markers, such as *EGR1*, *DCN*, *PRELP*, *FMOD* and *COMP*, which have all been shown to be involved in tenogenic regulation [51]. Interestingly, quite a number of *MMPs* were upregulated and their inhibitors *TIMPs* were downregulated in the tECM group compared with Col1 group, indicating high matrix remodeling activity [15]. Unexpectedly, growth factors and their receptors whose activities have been best characterized during tendon healing, such as FGFs, PDGF, IGFs, TGF-*β* and BMP2, did not show enhanced expression in the tECM compared with Col1 group [6]. Taken together, this expression profile suggests that the pro-tenogenic bioactivity of tECM may not involve these growth factor signals, but instead the synergistic contribution from multiple key ECM components.

## Conclusions

In summary, in this investigation we have characterized the protein composition of tECM as well as analyzed its pro-tenogenesis bioactivity and compared the transcriptome expression profiles of tECM- and Col1-treated hASCs. Our findings showed that urea-extracted tECM retained some collagens (~ 20% w/w) and were significantly enriched in many other lower molecular weight, non-collagenous ECM components. Compared to Col1 treated hASCs, tECM enhanced hASC tenogenic differentiation and exhibited distinct gene expression profiles. Thus, our tECM preparation represents an effective and highly reproducible approach of extracting bioactive ECM components from tendon and is a practically useful method for promoting tenogenesis. Additionally, the findings from this study provide essential clues into the potential mechanism action of tECM on tenogenic differentiation and thus present a rational basis for the application of tECM in tendon healing and regeneration.

## Supplementary Information

**Additional file 1:** Supplementary materials and methods.

## Data Availability

The datasets supporting the conclusions of this article are available from the corresponding author upon reasonable request.

## Abbreviations

ACN: Acetonitrile
BMP2: Bone morphogenetic protein 2
BSA: Bovine serum albumin
cDNA: Complementary DNA
CFU-F: Colony-forming unit-fibroblast
Col1: Collagen type I
COMP: Cartilage oligomeric matrix protein
CTGF: Connective tissue growth factor
DAPI: 4′, 6-Diamidino-2-phenylindole
DAVID: Database for Annotation, Visualization and Integrated Discovery
DEG: Differentially expressed genes
DMEM: Dulbecco's modified Eagle's medium
DNase: Deoxyribonuclease
DTT: Dithiothreitol
ECM: Extracellular matrix
FBS: Fetal bovine serum
FDA: Food and Drug Administration
FDR: False discovery rate
FGF: Fibroblast growth factor
GO: Gene Ontology
GSEA: Gene set enrichment analysis
GuHCL: Guanidine hydrochloride
hASC: Human adipose-derived stem cell
IAA: Iodoacetamide
IGF: Insulin-like growth factor
ITS-X: Insulin-transferring-selenium-ethanolamine
KEGG: Kyoto Encyclopedia of Genes and Genomes
LDET: Long digital extensor tendons
MALDI: Matrix-assisted laser desorption/ionization
MS: Mass spectrometry
MSC: Mesenchymal stem cell
NanoLC: Nanoflow liquid chromatography
P/S: Penicillin/streptomycin
PANTHER: Protein ANalysis THrough Evolutionary Relationships
PBS: Phosphate-buffered saline
PCC: Pearson correlation coefficient
qPCR: Real-time polymerase chain reaction
PDGF: Platelet-derived growth factor
PVDF: Polyvinylidene fluoride
RNA-Seq: RNA sequencing
RNase: Ribonuclease
SCX: Scleraxis
SD: Standard deviation
SDFT: Superficial digital flexor tendon
SDS-PAGE: Sodium dodecyl sulfate-polyacrylamide gel electrophoresis
TCEP: Tris [2-carboxyethyl] phosphine
tECM: Tendon-derived extracellular matrix
TGF-*β*: Transforming growth factor-beta
TNC: Tenascin C
TOF: Time of flight
TSP: Thrombospondin
TSPC: Tendon stem/progenitor cell
WJMSC: Wharton’s Jelly-derived MSC
