# Supplementary material for: Tenogenic induction of human adipose-derived stem cells by soluble tendon extracellular matrix: composition and transcriptomic analyses
Source: Stem Cell Res Ther. 2022 Jul 29;13:380. doi: 10.1186/s13287-022-03038-0 (PMC9338462; doi:10.1186/s13287-022-03038-0)
Supplement: Supplementary file 1 — Additional file 1: Supplementary materials and methods. [file 13287_2022_3038_MOESM1_ESM.docx]

**Tenogenic Induction of Human Adipose-derived Stem Cells**

**by Soluble Tendon Extracellular Matrix: Composition and Transcriptomic Analyses**

Ying Rao^1,2^, Chenxian Zhu^1,2^, Hoi Ching Suen^2^, Shuting Huang^1,2^, Jinyue Liao^5,2^,

Dai Fei Elmer Ker^1,2,3,4,6^, Rocky S. Tuan^1,2,6*^, Dan Wang^1,2,3,4,6*^

**Supplementary file**

**Materials and Methods**

**MS analysis and protein identification**

The peptide mixtures from the in-gel digestion procedure were loaded onto a column (Thermo Fisher Scientific, Acclaim^TM^ PepMap^TM^ 100 C18; length:150 mm, diameter: 0.075 mm, particle size: 2 µm) and separated using a segmented gradient within 7 min from 5%–35% of ACN at a flow rate of 300 nL/min. 50nL of sample eluant was automatically mixed with 420 nL of matrix solution α-cyano-4-hydroxycinnamic acid (HCCA) and spotted onto a MALDI target plate (AnchorChip, Bruker Daltonics, Germany). The whole MALDI-TOF/TOF system was controlled by the HyStar 3.2 software (Bruker Daltonics). The MS spectra were externally calibrated using a Peptide Calibration Standard II (Bruker Daltonics). A list of precursor peaks was obtained using the WARP-LC software (Bruker Daltonics). The chosen discriminative mass-to-charge ratio (m/z) was analyzed with MS/MS mode for protein identification. Proteins were identified from their peptide mass fingerprint by using Proteinscape (v. 3.1, [https://www.medicalexpo.com](https://www.medicalexpo.com/)) against the SwissProt database (<http://expasy.org/sprot/>) and Mascot 2.4.1 search engine (Matrix Science, London, UK) with taxonomical restriction to “Other mammalian”. The following general protein search parameters were used: 50 ppm mass tolerance for precursor ions, fragment ion mass tolerance of 0.7 Da, enzyme search for trypsin, modifications were oxidation and carbamidomethyl [1]. Protein categorization was performed using annotations from The Matrisome Project [2] (In silico Matrisome [http://matrisomeproject.mit.edu](http://matrisomeproject.mit.edu/)) and the Protein ANalysis THrough Evolutionary Relationships (PANTHER) classification system [3] (v. 16.0 <http://pantherdb.org>). Gene ontology (GO), signalling pathway and protein network analyses of identified proteins were performed using the Search Tool for Retrieval of Interacting Genes/Proteins (STRING, v. 11.5, <http://geneontology.org>). [4]

**RNA-Seq analysis**

Samples were sequenced by the Novaseq 6000 system (Illumina, USA) using approximately 150 base-pair paired-end RNA-Seq technology with 60-90 million reads per sample. Data were analyzed using bioinformatics software, and raw sequencing reads trimmed by Trim Galore (v. 0.6.6, [http:/www.bioinformatics.babraham.ac.uk/projects/trim_galore/](http://www.bioinformatics.babraham.ac.uk/projects/trim_galore/)) [5], were mapped to a human reference genome (Homo_sapiens. GRCh38). A transcriptome assembly was generated for each sample, and expression levels were calculated for each transcript or gene with read counts and transcripts per million (TPM) by using the Kallisto software (v. 0.46.1, <https://pachterlab.github.io/kallisto/>) [6]. Gene expression analysis was carried out using the program R (v. 3.6.1, <http://www.r-project.org/>) and data presentation was performed using R packages, including edgeR (v. 3.28.1) [7] and DESeq2 (v. 1.26.0) [8]. Expression correlation was calculated using Pearson’s r coefficient with pairwise-complete correlation method (generated by R packages) as described previously [5]. Genes with > 2-fold change or false discovery rate (FDR) < 0.05 were considered as differentially expressed genes (DEG) and visualized by volcano plots via GraphPad Prism v. 8.4.2 (GraphPad Software, USA, [https://www.graphpad.com](https://www.graphpad.com/)) as well as heatmaps via the Heatmapper [9] (Canada, <http://www.heatmapper.ca/>). Gene Ontology (GO) analysis was performed using the PANTHER classification system [10] (<http://geneontology.org>), with an FDR < 0.05 being regarded as significantly enriched. Pathway analysis was performed by using the Kyoto Encyclopedia of Genes and Genomes (KEGG) in the Database for Annotation, Visualization and Integrated Discovery [11] (DAVID, Resources 6.8, <https://david.ncifcrf.gov>) with an FDR < 0.05 being regarded as significantly enriched. Gene set enrichment analysis (GSEA; v. 4.1.0, <https://www.gsea-msigdb.org/gsea/index.jsp>) was performed to examine the signiﬁcantly enriched KEGG pathways. T-test was used as a metric for ranking genes to the GSEA Molecular Signatures Database (MsigDB). All the annotated transcripts (around 36000 features in total) with TPM expression values were uploaded to a locally installed GSEA tool and compared against the catalogue C2 KEGG subset of Canonical pathways (186 curated human gene sets). Gene sets were only considered significantly enriched if the absolute value of normalized enrichment score (NES) > 1, *P* < 0.05, and FDR < 0.25 [12].

**References:**

1. Swiatly A, Horala A, Hajduk J, Matysiak J, Nowak-Markwitz E, Kokot ZJ. MALDI-TOF-MS analysis in discovery and identification of serum proteomic patterns of ovarian cancer. BMC Cancer. 2017;17(1):472.

2. Hynes RO, Naba A. Overview of the matrisome--an inventory of extracellular matrix constituents and functions. Cold Spring Harb Perspect Biol. 2012;4(1):a004903.

3. Thomas PD, Kejariwal A, Campbell MJ, Mi H, Diemer K, Guo N, et al. PANTHER: a browsable database of gene products organized by biological function, using curated protein family and subfamily classification. Nucleic Acids Res. 2003;31(1):334-41.

4. Szklarczyk D, Gable AL, Lyon D, Junge A, Wyder S, Huerta-Cepas J, et al. STRING v11: protein-protein association networks with increased coverage, supporting functional discovery in genome-wide experimental datasets. Nucleic Acids Res. 2019;47(D1):D607-13.

5. Liao J, Ng SH, Luk AC, Suen HC, Qian Y, Lee AWT, et al. Revealing cellular and molecular transitions in neonatal germ cell differentiation using single cell RNA sequencing. Development. 2019;146(6):dev174953.

6. Bray NL, Pimentel H, Melsted P, Pachter L. Near-optimal probabilistic RNA-seq quantification. Nat Biotechnol. 2016;34(5):525-7.

7. Robinson MD, McCarthy DJ, Smyth GK. EdgeR: a Bioconductor package for differential expression analysis of digital gene expression data. Bioinformatics. 2010;26(1):139-40.

8. Anders S, Huber W. Differential expression analysis for sequence count data. Genome Biol. 2010; 11(10):R106.

9. Babicki S, Arndt D, Marcu A, Liang Y, Grant JR, Maciejewski A, et al. Heatmapper: web-enabled heat mapping for all. Nucleic Acids Res. 2016;44(W1):W147-53.

10. Mi H, Muruganujan A, Casagrande JT, Thomas PD. Large-scale gene function analysis with the PANTHER classification system. Nat Protoc. 2013;8(8):1551-66.

11. Dennis G Jr, Sherman BT, Hosack DA, Yang J, Gao W, Lane HC, et al. DAVID: database for annotation, visualization, and integrated discovery. Genome Biol. 2003;4(5):P3.

12. Subramanian A, Kuehn H, Gould J, Tamayo P, Mesirov JP. GSEA-P: a desktop application for Gene Set Enrichment Analysis. Bioinformatics. 2007;23(23):3251-3.
